# Special Issue: “Actinobacteria and Myxobacteria—Important Resources for Novel Antibiotics”

**DOI:** 10.3390/microorganisms8101464

**Published:** 2020-09-24

**Authors:** Joachim Wink

**Affiliations:** Microbial Strain Collection, Helmholtz Centre for Infection Research GmbH (HZI), Inhoffenstrasse 7, 38124 Braunschweig, Germany; Joachim.Wink@helmholtz-hzi.de

The history of our antibiotics is inseparably connected to microorganisms as producers. In particular, microorganisms with large genomes (often more than 8 MB) like many Actinobacteria and the *Myxococcales* show the highest potential for secondary metabolite formation. In addition, an important factor seems to be the differentiation process which is also found in both of these bacterial groups. Many of the clinically used antibiotics like the cephalosporins, anthrycylines, macrolides, glycopeptides, lipopetides and aminoglycosides are originally products of Actinobacteria [1]. The producers belong to a number of different genera like *Streptomyces*, *Amycolatopsis*, *Micromonospora* and *Actinoplanes.* The phylum Actinobacteria belongs to the Gram-positive bacteria with a high GC content. They can be mainly found in soil but there are also pathogenic and saprophytic species. In particular, the mycelium-forming genera show characteristic differentiation by forming endospores that can be arranged in spore chains or sporangia.

Besides, the Actinobacteria members of the *Myxococcales* were first reported to show bacteriolytic effects in 1946 [2] but it took until the 1980s for the first antibiotic with high potential for market development, Sorangicin, to be isolated [3]. Like Actinobacteria, most of the members of the *Myxococcales* live in soil, they belong to the Gram-negative bacteria and form fruiting bodies during their differentiation process. With the knowledge of the genome information, it is now clear that they also harbor a large potential for the production of secondary metabolites [4,5].

The isolation of novel Actinobacteria and Myxobacteria still leads to new genetic potential for the identification and isolation of bioactive compounds, especially antibiotics. With more and more understanding of this genetic information, we also see a huge number of genes for which we do not know the resulting product and the induction of these silent genes is one of the challenges [6].

This issue gathers 16 papers including 11 articles, 4 reviews and 1 communication. Six of them describe novel species or isolates and their characterization by the use of a polyphasic approach [7,8,9,10,11,12] as well as their secondary metabolites. Two of the articles describe Actinobacteria from uncommon habitats like the Western Ghats region in India [13] and endophytic ones [14]. The study of the induction of secondary metabolites by use of the OSMAC approach is the subject of one other article [15]. The other two articles include the identification and heterologous expression of an antibiotic gene cluster [16] and the biological activity of some volatile secondary metabolites [17]. In the communication, the authors report the unexplored biosynthetic potential in Myxobacteria [18]. In the review section, we find reports on polyketide biosynthesis in *Streptomyces* [19], the role of elicitors in antibiotic biosynthesis [20], antiviral compounds from Myxobacteria [21] and an overview on the role of Myxobacteria as secondary metabolite producers [22]. Altogether, in my view, this is a balanced snapshot of this impressive research field.
